# A Drug Repurposing Screen Identifies Fludarabine Phosphate as a Potential Therapeutic Agent for N-MYC Overexpressing Neuroendocrine Prostate Cancers

**DOI:** 10.3390/cells11142246

**Published:** 2022-07-20

**Authors:** Hussain Elhasasna, Raymond Khan, Kalpana K. Bhanumathy, Frederick S. Vizeacoumar, Prachi Walke, Maricris Bautista, Dinesh K. Dahiya, Vincent Maranda, Hardikkumar Patel, Amrutha Balagopal, Nezeka Alli, Anand Krishnan, Andrew Freywald, Franco J. Vizeacoumar

**Affiliations:** 1 Division of Oncology, College of Medicine, University of Saskatchewan, Saskatoon, SK S7N 5E5, Canada; hussain.hasasna@usask.ca (H.E.); khanraymond@hotmail.com (R.K.); kalpana.kbk@gmail.com (K.K.B.); frederick.vizeacoumar@usask.ca (F.S.V.); prachi.walke@usask.ca (P.W.); euz140@mail.usask.ca (D.K.D.); qay582@mail.usask.ca (V.M.); qvs729@mail.usask.ca (H.P.); amb850@mail.usask.ca (A.B.); nezeka@gmail.com (N.A.); 2 Department of Pathology, College of Medicine, University of Saskatchewan, Saskatoon, SK S7N 5E5, Canada; 3 Department of Anatomy, Physiology, and Pharmacology, University of Saskatchewan, and Cameco MS Neuroscience Research Centre, 701 Queen St., Saskatoon, SK S7K 0M7, Canada; maricris.bautista@usask.ca (M.B.); anand.krishnan@usask.ca (A.K.); 4 Cancer Research Department, Saskatchewan Cancer Agency, 107 Wiggins Road, Saskatoon, SK S7N 5E5, Canada

**Keywords:** NEPC, fludarabine phosphate, drug repurposing, MYCN

## Abstract

Neuroendocrine prostate cancer (NEPC) represents a highly aggressive form of prostate tumors. NEPC results from trans-differentiated castration-resistant prostate cancer (CRPC) with increasing evidence indicating that the incidence of NEPC often results from the adaptive response to androgen deprivation therapy. Recent studies have shown that a subset of NEPC exhibits overexpression of the *MYCN* oncogene along with the loss of tumor suppressing TP53 and RB1 activities. N-MYC is structurally disordered with no binding pockets available on its surface and so far, no clinically approved drug is available. We adopted a drug-repurposing strategy, screened ~1800 drug molecules, and identified fludarabine phosphate to preferentially inhibit the proliferation of N-MYC overexpressing NEPC cells by inducing reactive oxygen species (ROS). We also show that fludarabine phosphate affects N-MYC protein levels and N-MYC transcriptional targets in NEPC cells. Moreover, enhanced ROS production destabilizes N-MYC protein by inhibiting AKT signaling and is responsible for the reduced survival of NEPC cells and tumors. Our results indicate that increasing ROS production by the administration of fludarabine phosphate may represent an effective treatment option for patients with N-MYC overexpressing NEPC tumors.

## 1. Introduction

Prostate cancer (PC) is the most common non-skin cancer among men and is the second leading cause of cancer-related deaths in men in North America [1]. Androgen deprivation therapy (ADT) is effective for treating PCs, however, disease remission is temporary, as the cancer resurfaces as castration-resistant PC (CRPC). Androgen receptor (AR) activation persists in CRPC, in part, due to the tumoral androgen synthesis [2,3,4]. While new generation anti-androgen drugs such as enzalutamide [5] and abiraterone [6] extend the survival of CRPC patients, resistance is inevitable, and in ~25–30% of resistant cases, CRPC trans-differentiates into NEPC, which leads to rapid lethality [7,8,9]. Thus, NEPC represents the hormone-refractory late manifestation of PC with no treatment options [10,11].

N-MYC is known to be among the key drivers of NEPC [12,13]. Aberrant amplification and/or overexpression of *MYCN* is not only observed in NEPC, but also in other aggressive tumors, such as neuroblastoma, medulloblastoma, retinoblastoma, small cell carcinoma of the lung, pancreatic neuroendocrine tumors, and glioblastoma to name a few [14,15,16]. In fact, Pan-cancer analyses of the tumor sequencing data from cancer genome atlas data, in 33 cancers, found that 28% of all samples had at least one of the MYC paralogs amplified [17]. With respect to PC, the MYC family of transcription factors are implicated in the development and progression of the full spectrum of PC, from adenocarcinoma to CRPC to NEPC [12,18,19,20,21,22,23,24]. Specifically, *MYCN* is amplified in 40% of NEPC cases and possibly to a similar extent in PCs with NEPC features [12,13,21,25]. *MYCN* amplification and upregulation have been linked to cancer through several mechanisms. For example, transcription factors such as SP1, E2F, and PLAGL2 have been shown to directly bind to the cognate binding sites in the *MYCN* promoter, contributing to *MYCN* activation [26,27,28]. In fact, the promoter region of *MYC* genes contains super-enhancer areas that are recognized by chromatin readers such as BRD4, which in turn recruits transcription factors, cofactors, and chromatin regulators, thereby promoting transcription activity [29]. Apart from enhanced transcription, tumor cells also maintain high MYC protein levels by evading protein turnover via the PI3K/AKT pathway [30,31]. Normally, N-MYC is degraded during mitosis, by the ubiquitin ligase FBXW7 via the ubiquitin–proteasome system, following N-MYC phosphorylation by cyclinB/CDK1 and glycogen synthase kinase-3β (GSK3β) [32]. However, when the PI3K/AKT pathway is activated in cancer cells, this results in GSK3β inactivation, thereby preventing N-MYC phosphorylation and degradation. MYC is also stabilized by Aurora kinase A (AURKA) which prevents its binding to FBXW7 [33]. The Polo-like Kinase 1 (PLK1) stabilizes MYC by phosphorylating and promoting proteasomal degradation of FBXW7 [34]. Thus, tumor cells promote *MYCN* expression and N-MYC protein levels via multiple mechanisms.

Disappointingly, previous efforts to directly inhibit MYC protein, including N-MYC with small-molecule inhibitors (SMIs) were not effective due to insufficient inhibition or activation of compensatory mechanisms [35,36,37,38]. Although direct inhibition of MYC is challenging due to the lack of conventional drug target sites [39], MYC elicits its oncogenic effects by forming a heterodimer with MAX [40,41] and disrupting this interaction provides a potential opportunity. SMIs including 10058-F4, 10074-A4, and 10074-G5 have been characterized as potential MYC-MAX binders [35,36,37,38]. Unfortunately, their derivatives, Mycro3 and KJ-Pyr-9, did not exhibit strong anti-tumor potency in vivo [36]. To address this, new approaches that aim to inhibit multiple molecules that are part of Myc signaling are also emerging. For example, dual inhibitors of BRD4 and PLK1 that prevent both *MYCN* transcription and promote N-MYC degradation, respectively, were found to exhibit antitumor effects in preclinical pediatric cancer models [42]. Similarly, a dual inhibitor of N-MYC and AURKA has also shown the potential effect on NEPC cells [43]. While these novel drugs are very promising, further optimization of these molecules is required, their toxicity needs to be evaluated and their effects on NEPC models are yet to be tested. As such, new therapeutic strategies to treat NEPC patients are still lacking.

Traditionally, new therapeutic compound development can take several years before they reach patients and require an enormous financial investment [44]. The rate-limiting step in drug discovery has always been extensive protocols entailed by clinical trials. In this context, drug repurposing strategies represent viable alternatives, allowing for the rapid identification of effective therapeutics. We undertook a drug-repurposing screening strategy that aimed at eliminating selectively *MYCN* overexpressing NEPC cells. We report here the identification of fludarabine phosphate as a potential agent that preferentially inhibits *MYCN*-overexpressing NEPC cells and tumors, in an ROS-dependent fashion.

## 2. Materials and Methods

### 2.1. Cell Lines Used in This Study

LASCPC-01 (ATCC^®^ CRL-3356) was from ATCC (Manssas, VA, USA). LASCPC-01 cells were cultured in modified HITES medium as described [45]. Briefly, HITES media containing RPMI, 5% FBS, 10 nM hydrocortisone, 10 nM beta-estradiol (Millipore Sigma, Burlington, MA, USA), insulin–transferrin–selenium, and Glutamax (Life Technologies, Carlsbad, CA, USA). HEK293T cells were cultured in DMEM (Hyclone, #SH30243.01, Logan, UT, USA) containing 10% FBS and supplemented with 1% penicillin/streptomycin. Fludarabine phosphate (#S1229, Selleckchem, Houston, TX, USA). N-acetyl-L-cysteine (A7250, Sigma), Bortizomeb (#S1013, Selleckchem). The cell line LNCaP, and LNCaP-N-MYC as well as 22Rv1, and 22Rv1-N-MYC cells were kindly provided by the Rickman Lab from Cornell Medical College. Cells were cultured in the RPMI 1640 medium (Hyclone, #SH30027.01) containing 10% Fetal Bovine Serum (FBS) (Hyclone, F1051), 1% penicillin/streptomycin (Hyclone, #SV30010) at 37 °C with 5% CO_2_.

### 2.2. Drug Screen Using Targetmol Drug Library

Targetmol Drug Library (Wellesley Hills, MA, USA) Stock concentration was 10 mM per Drug in DMSO. Each drug was diluted into 384-well plates to target concentrations with dimethyl sulfoxide (Fisher BioReagents #L-15579, Pittsburg, PA, USA) as per Targetmol instructions. A total of 2.5 μL of 10 mM was combined with 47.5 μL of dimethyl sulfoxide (Fisher BioReagents #L-15579) to yield 50 μL of 500 μM per drug plate. Then, 16.25 μL of 500 μM was combined with 16.25 μL of dimethyl sulfoxide (Fisher BioReagents #L-15579) to yield 32.5 μL of 250 μM per drug plate. Then, 12.5 μL of 250 μM was combined with 12.5 μL of dimethyl sulfoxide (Fisher BioReagents #L-15579) to yield 25 μL of 125 μM per drug plate. Finally, 5 μL of 125 μM was combined with 20 μL of dimethyl sulfoxide (Fisher BioReagents #L-15579) to yield 25 µL of 25 μM. Plates sealing film was used to seal the 24 plates (6 plates per concentration) and plates were stored at −20 °C. Target concentration after 200 nL dispensed from plates are: 1000 nM, 500 nM and 100 nM. LNCaP-RFP and LNCaP-N-MYC- RFP were counted using CountessTM II Automated Cell Counter (#AMQAX1000) and cell lines were plated at a density of 300 cells/well in 50 μL of in RPMI 1640 (Hyclone, #SH30027.01) containing 10% fetal bovine serum (Hyclone, #F1051) supplemented with 1% penicillin/streptomycin (Hyclone, #SV30010). Each cell line was plated in 6384-well imaging plates (#142761) using a BioMek NX liquid handling robot. Cell count was assessed via Molecular Devices XLS non-confocal high throughput microscope (San Jose, CA, USA) prior to treatment with Targetmol Drug Library. Cell distribution found 95% of wells were plated within 15% of mean of all wells, which was congruent with the high degree of accuracy expected from automated liquid handling robots. Drug pinning tool by V&P Scientific (San Diego, CA, USA) was used by BioMek NX liquid handling robot to dispense 200 nL from drug plate to 384-well imaging plates (#142761) containing each cell line; LNCaP-P-RFP and LNCaP-MYCN-RFP. Drug pinning tool by V&P Scientific was cleaned prior to each cell line treatment by washing in double distilled water, then drying with lint-free drying paper, then washing with 100% ethanol, and finally drying again with another piece of lint-free drying paper. Drugs were dispensed 96 wells at a time, with cleaning protocol with double distilled water and 100% ethanol performed between dispensing.

### 2.3. High-Throughput Microscope Imaging

LNCaP-P-RFP and LNCaP-MYCN-RFP were plated at a density of 300 cells/well in 384-well imaging plates (#142761) and plates were imaged using non-confocal high throughput microscope by Molecular Devices. The benefits of using high-throughput imaging allowed for detection of the onset of action as well as the duration of action via monitoring cell count. Images were taken at 4× magnification, with 4 images taken per well. Automated cell counts were assessed via MetaXpress software (Molecular Devices, San Jose, CA, USA) with Texas Red filter and count was optimized via digital and manual count.

### 2.4. Computational Scoring of Drug Library Screen

Growth inhibition was determined by calculating the B score [46] of the cell counts. The cell counts were generated based on the MetaXpress image analysis software of the high-throughput microscope. B score is the ratio of adjusted raw value to measure of variability [47]. The adjusted raw value accounts for positional effects as well as plate-to-plate changes in the means, while the variability is a resistant measure of the residual variability in the plates after plates are fitted by median polish by using median absolute deviation for each plate. The residual is calculated by the difference between observed results and fitted values. The fitted value is the sum of the estimated average of the plate, measurement offsets of row and column on that plate. We calculated the B scores of the LNCaP and LNCaP-N-Myc lines to compare them across various time points and concentrations.

### 2.5. IncuCyte Live Cell Analysis

Cell proliferation over time was monitored using an IncuCyte Analysis S3 System and software (Sartorius, Gottingen, Germany). LASCPC01 cells were seeded in 96-well plates at 10^4^ cells/well and the confluency of each well was measured every 8 h, over the course of 5 days.

### 2.6. SDS-PAGE and Western Blot Analysis

LASCPC-01 cells were seeded at 2–2.3 × 10^6^ cells per 10 cm plate, post-fludarabine phosphate treatment. Pre-treatment for 3 h with or without NAC at10 mM and pre-treatment with or without Bortezomib at 5 nM followed by fludarabine phosphate treatment at concentration of 25 µM cells were collected at 48 h and total cell lysates were prepared using RIPA buffer (Pierce, 89901). Protein lysates were collected, and the concentration of the protein was quantified using a bicinchoninic acid (BCA) assay (Thermo Scientific, 23225, Waltham, MA, USA). Equal amounts of protein (15–30 µg) were loaded and electrophoresed on a sodium dodecyl sulfate (SDS) polyacrylamide gel (PAGE) 10% in Laemelli buffer, followed by wet transfer to NC membranes (BioRad, 162-0177, Hercules, CA, USA). Membranes were blocked with 5% skimmed milk in 0.1% Tween-20 in Tris-buffered saline (TBST) for 1 h at room temperature. The membranes were then incubated with the primary antibodies at a dilution of 1: 1000 at 4 °C overnight. The next day, membranes were washed 3 times with TBST 5 min each and incubated for 1 h with peroxidase-conjugated secondary antibody at a dilution of 1: 5000 for 1 h at room temperature. After washing three times with TBST, the protein bands were visualized using Clarity™Western ECL chemiluminescent substrate (BioRad, 170-5060) and ChemiDoc™ MP Imaging System (BioRad) following the manufacturer’s procedure. The brightness of Western blot images was adjusted where required, using power point. However, original unedited full-length Western blots are provided in the Supplementary Figures S4 and S5. Image Lab™ version 6.0.0 build 25 software were used to perform the bands densitometry. The following antibodies were obtained from Santa Cruz: N-Myc (sc-53993), GAPDH (sc-25778). Cell Signaling: AKT #9272, phospho-AKT Ser473 #9271, pMEK 1/2 (Ser 217/221) #9154, SYP #36406, Caspase-3 #9662. From Millipore H2A.X (Ser139) 05-636, NSE MAB324. Beta III Tubulin (AB9354, Sigma-Aldrich). Beta Tubulin (Abcam, ab15568, Cambridge, UK). Secondary antibodies: Anti mouse-HRP #7076, Anti Rabbit-HRP #7074, Anti-chicken-HRP invitrogen A16054.

### 2.7. qRT-PCR

Total RNA was isolated using TRIzol™ reagent (Life Technologies 15596018) and quantified using NanoDrop^®^ ND-1000 Spectrophotometer (Thermo Fisher). Afterward, cDNA was synthesized from one microgram of total RNA using High-Capacity cDNA Reverse Transcription Kit (Applied Biosystems 4368813) according to the manufacturer’s instructions. The primers used to amplify the cDNAs are in (Supp. Table S2) The amplifications were completed using PowerUp™ SYBR™ Green Master Mix (Applied Biosystems A25741) and performed in QuantStudio™ 3 Real-Time PCR System (Applied Biosystems, Waltham, MA, USA). Cycling conditions are 40 cycles with denaturation at 95 °C for 10 s. Annealing and extension were performed at 60 °C for 20 s to promote primer binding to the template and subsequent elongation occurs due to sufficient activity of the DNA polymerase at this temperature. All reactions were carried out in triplicate. The mRNA expressions were normalized to the housekeeping mRNAs, RPLP or GAPDH.

### 2.8. RNASeq Analysis

The LASCPC-01 control and treated samples were sequenced using Illumina HiSeq 4000 according to manufacturer’s instructions. Total RNA was extracted from the cell pellets using Qiagen RNeasy Plus Universal mini kit (Qiagen, Hilden, Germany). Quantification of the extracted RNA samples was performed using Qubit 2.0 Fluorometer (Life Technologies, Carlsbad, CA, USA) and RNA integrity was verified using Agilent TapeStation 4200 (Agilent Technologies, Palo Alto, CA, USA). Briefly, mRNAs were first enriched for Oligo(dT) beads. Enriched mRNAs were fragmented for 15 min at 94 °C. Subsequently, first and second-strand cDNAs were synthesized. Next, we performed end repair and 3′end adenylation of the cDNA fragment and ligated the universal adapter to the cDNA fragment. Finally, added the index and enriched the library through limited cycle PCR. The sequencing library was verified on Agilent TapeStation (Agilent Technologies, Palo Alto, CA, USA) and quantified using Qubit 2.0 fluorometer (Invitrogen, Carlsbad, CA) and quantitative PCR (KAPA Biosystems, Wilmington, MA, USA). The samples were sequenced using 2 × 150 bp paired-end configuration by Genewiz (South Plainfield, NJ 07080, USA). Image analysis and base calling were conducted by the HiSeq Control Software. Raw sequence data (.bcl files) generated from Illumina HiSeq was converted into fastq files and demultiplexed using Illumina’s bcl2fastq v.2.16. One mismatch was allowed for index sequence identification. The sequence reads were then trimmed to remove adapter sequences and poor-quality nucleotides using Trimmomatic v.0.36. These trimmed sequences were mapped to GRCh38 reference genome (Homo sapiens) from ENSEMBL using STAR aligner v.2.5.2b. These STAR aligner detects splice junctions and uses them to align the entire read sequences to generate BAM files. We then used these BAM files to generate unique gene hit counts using featureCounts from the Subread package v.1.5.2. These counts were summarized using the gene_id feature in the annotation file. The unique read counts from the exon regions were counted. Upon extraction of the gene counts, we used DESeq2 R package [48] to compare the control and treated samples to identify the differentially expressed genes. The Wald test was used to generate the p-value and log2 fold changes. The RNA seq data are deposited in Gene Expression Omnibus (GEO) database and can be obtained with the accession number GSE181119.

### 2.9. Xenograft Studies

All animal experimental procedures were reviewed and approved by the University of Saskatchewan Animal Research Ethics Board. Mice used in the present study were from our established colony of immunodeficient male NOD.Cg-*Prkdc^scid^ Il2rg^tm1Wjl^*/SzJ (NOD-SCID) mice at the Laboratory Animal Services Unit (LASU), University of Saskatchewan. Cells were trypsinized and re-suspended in ice-cold PBS. Cells were mixed 1: 1 with Matrigel (Corning, CB-40234), and 1 × 10^6^ cells in a total volume of 100 μL were injected subcutaneously into the right flank of 6 to 8 weeks old NOD/SCID mice. Where indicated, upon tumor development, intraperitoneal (i.p.) injections of fludarabine phosphate at 120 mg/kg in DMSO or a matching DMSO volume per injection, per day, were administered with five days of i.p injections, followed by two days of rest with no injections and then, continued with five more i.p. injections in five days. Tumors were measured every three days using a digital caliper, and the tumor volume was calculated using the tumor ellipsoid formula A/2*B^2^, where A and B represent the long and the short diameter of the tumor, respectively. Upon experiment termination, tumors were extracted for immunohistochemistry and Western blotting. For Western blotting, tumor tissues were homogenized using tissue extraction lysis buffer (Thermo Scientific, 87792), and protein quantification were performed using the BCA kit (Thermo Scientific, 23225).

### 2.10. Immunohistochemistry

Prostate tumor samples isolated from mice were fixed overnight at 4 °C using Zamboni’s fixative (2% paraformaldehyde and 0.5% picric acid in 1× PBS). Then, the samples were washed thrice in PBS and cryoprotected using 20% sucrose overnight at 4 °C. After three PBS washes, the tissues were embedded in Tissue-Plus™ O.C.T. Compound. Twelve µm thick sections were then taken on slides and the sections were blocked using 5% donkey serum containing 0.3% Triton X-100 for 30 min. The sections were afterward incubated for 1 h at room temperature (RT) with one of the following primary antibodies: mouse anti-Chromogranin A Clone DAK-A3 (1:100, Agilent DAKO M0869, Santa Clara, CA, USA), rabbit anti-Chromogranin B (1:100, Invitrogen PA5-52605, Waltham, MA, USA), or rabbit anti-Synaptophysin (D8F6H) XP^®^ (1:100, Cell Signaling 36406, Danvers, MA, USA). Afterward, the sections were incubated with either goat anti-mouse Alexa Fluor 546 (Invitrogen A21045) or goat anti-rabbit Alexa Fluor 546 (Invitrogen A11035) for 1 h in the dark at RT. The sections were mounted using SlowFade™ Diamond Antifade Mountant with DAPI (Invitrogen S36973), and all images were captured using an Axio Observer 7 inverted fluorescence microscope (Carl Zeiss, Oberkochen, Germany).

### 2.11. Statistical Analysis

Data were expressed as mean ± SD and *p*-values calculated with Student’s *t*-test. *p*-values < 0.05 were considered significant, with *p* values < 0.05, <0.01, and <0.001 indicated as *, **, ***, respectively.

## 3. Results

### 3.1. A Repurposing Drug Library Screen Identifies Compounds That Selectively Inhibit the Growth of N-MYC Overexpressing Prostate Cancer Cells

**Figure 1:**
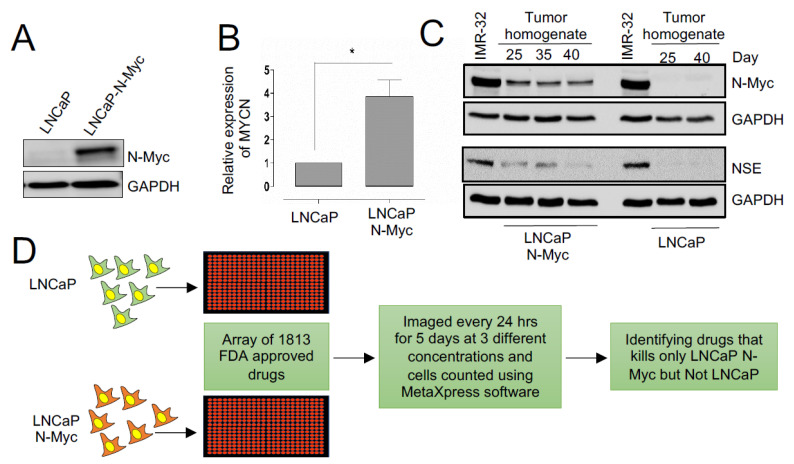
Identifying drugs with differential effects on the fitness of N-Myc overexpressing LNCaP cells. (**A**) Western blot showing N-MYC protein levels in the indicated isogenic cell lines. GAPDH is used as a loading control. (**B**) Expression levels of *MYCN* tested by RT-PCR in the isogenic cell lines (*p* < 0.05). (**C**) Western blots of LN-P and LN-Myc tumor homogenates at the indicated time points post cell implantation. Levels of N-MYC and NSE were assessed. (**D**) Schematic representing the pipeline of the high-throughput drug library screening in isogenic models. An array of FDA-approved drug libraries was used to identify those compounds that inhibit the growth of LNCaP-N-Myc but not the LNCaP cells. * *p* < 0.05.

To identify drugs capable of inhibiting the proliferation of cells overexpressing N-MYC, we used an isogenic cell line model, LNCaP-Parental (LN-P) and LNCaP-N-Myc (LN-Myc), where only LN-Myc cells overexpress N-MYC [13] (Figure 1A,B). The differential expression of N-MYC in this model has been previously shown to confer differential tumorigenic behavior, demonstrating the impact of N-MYC overexpression on tumorigenesis [13]. Prior to performing our initial screen, we confirmed the ability of these cells to form tumors and assessed N-MYC levels in tumor lysates (Figure 1C). We also evaluated the expression of neuron-specific enolase (NSE) and found that only LN-Myc tumors and not LN-P tumors express NSE, like a previously described N-MYC expressing neuroblastoma cell line model, IMR-32 (Figure 1C). Thus, LN-P and LN-Myc cells, represent an ideal isogenic model for performing our drug screen to identify compounds that preferentially inhibit the proliferation of N-MYC overexpressing cells (Figure 1D). Therefore, we used LN-P and LN-Myc cells to screen the Targetmol Drug Library comprised of 1813 drugs; 61% were approved by the FDA, and 38% were clinically used outside the United States (Supp. Figure S1A). A small percentage of these drugs have been withdrawn from clinical use due to adverse side effects or the availability of better alternatives. Approximately 50 compounds in the list are at different phases of clinical trials. The list contains small molecules that target a variety of conditions, including cardiovascular and respiratory diseases, and chemotherapeutic agents (Supp. Figure S1A). There are currently 143 FDA-approved small molecules in the drug library that target different human malignancies, with the majority targeting hematological cancers and the most common mechanism of action being DNA/RNA synthesis inhibition. Until now, this drug library has not been used to examine therapeutic effects in NEPC models.

**Figure 2:**
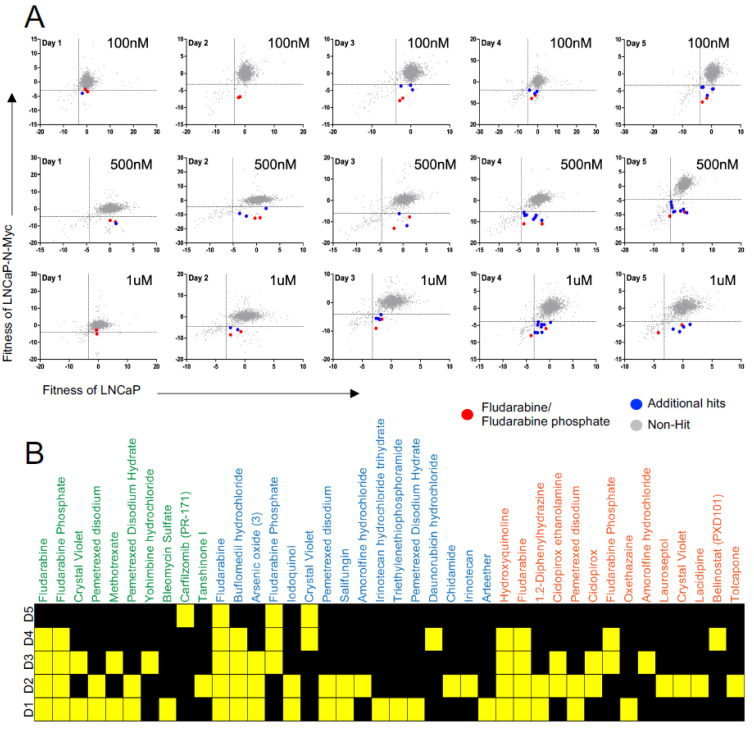
High-throughput drug library screen identified potential lead compounds that selectively inhibits proliferation *MYCN* overexpressing cells. (**A**) Scatter plot graphs show that fludarabine/fludarabine phosphate is a lead compound eliminating *MYCN* overexpressing cells. FDA-approved drug library compounds were applied at three different concentrations: 100 nM top panel, 500 nM middle panel, and 1 mM bottom panel to LNCap (*x*-axis) and LNCap-N-Myc (*y*-axis) cells and the cells were monitored for five days using high content imaging. Fludarabine/fludarabine phosphate red dots, additional hits blue dots, and non-hit grey dots. (**B**) Heatmap plot shows fludarabine and fludarabine phosphate exhibit preferential inhibition of LNCap-N-Myc cells at 100 nM, 500 nM, and 1 mM concentrations over five days (D1, D2, D3, D4, and D5). Yellow squares represent the days, where significant differences were identified. Hits in green text at 100 nM, hits in blue text at 500 nM, and hits in orange text at 1 mM (*p* < 0.05).

To monitor the response of NEPC cells, we transduced LN-P and LN-Myc cells with a lentivirus expressing RFP and we treated them with these drugs at three different concentrations (100 nM, 500 nM and 1 μM) and monitored them for five days using a high content imaging platform. Unlike end-point assays, this time course-dependent monitoring allowed us to continuously capture the response of live cells over several days with different drug concentrations (Figure 2A). This approach identified several molecules with differential effects on the isogenic cells, including DNA synthesis inhibitors such as irinotecan, fludarabine, fludarabine phosphate, daunorubicin; folate antimetabolites such as methotrexate, pemetrexed; proteasome inhibitor carfilzomib; HDAC inhibitors such as belinostat, chidamide, and antifungal agents such as hydroxyquinoline, salifungin, and ciclopirox (Figure 2B; Supp. Table S1). Daunorubicin or chidamide has previously been shown to affect C-MYC levels in breast cancer models and acute myeloid leukemia (AML) cells, respectively [49,50]. Similarly, an antifungal agent and histone demethylase, ciclopirox, has been reported to inhibit N-MYC signaling in neuroblastomas [51]. Additionally, the HDAC inhibitor Belinostat was also found to decrease c-Myc expression in the rhabdomyosarcoma [52]. These results indicate that our screening approach has captured several promising lead candidates that might regulate the MYC family through distinct mechanisms. Among the hits, we found fludarabine and fludarabine phosphate to preferentially inhibit the proliferation of LN-Myc cells compared to LN-P cells at all the three concentrations tested apart from conferring sensitivity at multiple time points (*p* < 0.05) (Figure 2A,B). Given that we have identified fludarabine as well as its phosphorylated form, fludarabine phosphate, with high confidence, we set out to validate this lead compound in NEPC models and explore the mechanism of its action.

### 3.2. Fludarabine Phosphate Causes Apoptotic Cell Death in N-MYC Overexpressing Cells by Inducing ROS

**Figure 3:**
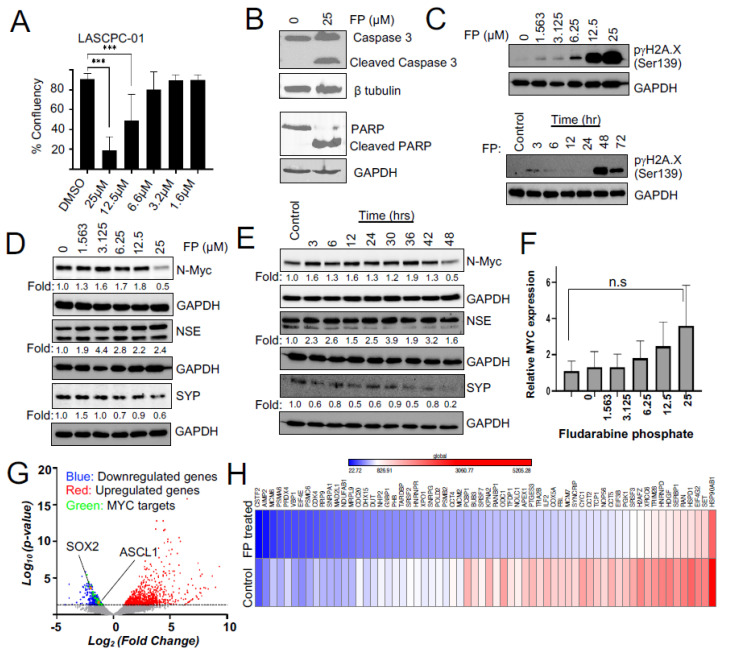
Fludarabine phosphate affects MYC protein levels and MYC transcriptional targets in NEPC cells. (**A**) Confluency measurements of LASCPC-01 cells following treatment of fludarabine phosphate 112 h after seeding (*** *p* < 0.001). (**B**) Fludarabine phosphate treatment induces apoptosis in LASCPC-01 cells at 25 μM. Western blot shows Figure 3 and PARP. b tubulin is used as a loading control. (**C**) Fludarabine phosphate causes DNA damage. Western blots show an increase in H2A.X in a dose-and-time-dependent manner in LASCPC-01 cells. Cells were treated with 25 μM fludarabine phosphate in the time course experiment. (**D**) Western blot shows a concentration-dependent decrease in N-MYC in fludarabine phosphate-treated LASCPC-01 cells. Cells were treated with DMSO or indicated concentrations of fludarabine phosphate for 48 h. Whole-cell extracts were subjected to Western blot analysis for N-MYC, NSE, and SYP. ImageLab software was used to quantify the bands with corresponding GAPDH and represented in the above blots. (**E**) Western blot shows time course analysis of N-Myc, NSE, and SYP levels at 48 h of 25 μM fludarabine phosphate treatment compared to the DMSO control at 48 h. (**F**) The e pression level of *MYCN* by RT-PCR in fludarabine phosphate-treated LASCPC-01 cells. Cells were treated with DMSO or indicated concentrations of fludarabine phosphate for 48 h. n.s not significant. (**G**) Volcano plot showing fold change in gene expression between fludarabine phosphate treated with 25 μM and untreated LASCPC-01 cells and their corresponding *p*-values at 48 h. (**H**) RNAseq expression analysis shows that 64 MYC-target genes were downregulated upon treatment with fludarabine phosphate in LASCPC-01 cells at 48 h compared to the DMSO-treated control.

We identified fludarabine phosphate and fludarabine as the top FDA-approved molecules that preferentially inhibited the proliferation of N-MYC overexpressing LN-Myc cells, compared to non-overexpressing parental cells. However, to eliminate cell line-specific effects, we also used an independent MYC-driven NEPC model, LASCPC-01 [45] in the validation experiments. We chose fludarabine phosphate for further studies as the addition of phosphate has been shown to enhance the solubility of the fludarabine [53], which could be beneficial for clinical applications. We found that N-MYC overexpressing LASCPC-01 cells are susceptible to fludarabine phosphate treatment at 12.5 µM and 25 µM (Figure 3A). Moreover, cleaved caspase 3 and cleaved PARP levels were consistently observed in the treated LASCPC-01 cells, suggesting that apoptotic cell death is a consequence of fludarabine phosphate application (Figure 3B). While the drug repurposing screen captured relative fitness differences at the indicated concentrations, the actual IC50 curves for LASCPC-01 are presented in Supp. Figure S1B.

Fludarabine phosphate is a purine analog and a prodrug that converts into its active triphosphate, 2-fluoro-ara-ATP form [54] before it gets incorporated into DNA to inhibit DNA synthesis [55]. Its incorporation into DNA may also affect the DNA repair [56,57]. Indeed, we found that fludarabine phosphate treatment elevated levels of γH2AX phosphorylated at serine 139 (Figure 3C) and this response is known to be triggered by DNA double-strand breaks, usually leading to a decrease in cell viability [58]. Taken together, these results suggest that fludarabine phosphate is likely to cause apoptotic cell death in N-MYC overexpressing cells.

### 3.3. Fludarabine Phosphate Affects N-MYC Protein Levels and N-MYC Transcriptional Targets in NEPC Cells

We next examined if fludarabine phosphate affected the expression and abundance of the N-MYC protein or neuronal markers in NEPC models. We monitored levels of the N-MYC protein after treating LASCPC-01 cells with increasing doses of fludarabine phosphate (Figure 3D) as well as over multiple time points (Figure 3E). We found that N-MYC protein levels increased at early time points and eventually dropped when LASCPC-01 cells were treated with 25 μM fludarabine phosphate for 48 h (Figure 3D,E). Interestingly, *MYCN* mRNA remained at a high level even at 25 μM of fludarabine phosphate (Figure 3F), indicating that the observed downregulation of N-MYC protein is not because of decreased mRNA production, but probably due to faster N-MYC protein turnover. Treatment with fludarabine phosphate, however, did not affect neuroendocrine marker NSE, while showing a decrease in the level of another marker, SYP (Figure 3D,E).

To further extend our analysis, we performed a whole transcriptome assessment by the RNA sequencing (RNAseq) of LASCPC-01 cells treated with 25 µM fludarabine phosphate for 48 h compared to untreated control cells (Figure 3G,H; Supp. Figure S2A,B). This identified 906 significantly upregulated and 662 downregulated genes in fludarabine phosphate-treated cells (Figure 3G). While the upregulated genes were enriched for extracellular matrix organization and cell junction organization, downregulated genes were enriched for Reactome pathways involved in the regulation of cell cycle and mitosis, cell cycle checkpoints, and DNA replication (Supp. Figure S2B). This is in line with the previous work suggesting a role for MYC in the cell cycle regulation [59]. Thus, N-MYC protein downregulation caused by fludarabine phosphate leads in turn, to the downregulation of several genes involved in cell cycle progression. While most neuronal markers were not affected, we found a slight decrease in the transcription factor that regulates neuronal differentiation (ASCL1; p 0.02159) and stem cell maintenance (SOX2; p 0.00023) (Figure 3G and Supp. Figure S2A). Interestingly, the RNAseq analysis also indicated that 64 MYC-target genes (MSigDb: M5926; https://www.gsea-msigdb.org/gsea/msigdb/cards/HALLMARK_MYC_TARGETS_V1 (accessed on 14 July 2021)) were downregulated upon treatment with fludarabine phosphate in LASCPC-01 cells (Figure 3G-green dots and Figure 3H). For example, MYC has been previously shown to influence mitotic spindle dynamics [60] and DNA replication [61], and consistent with this role, we found spindle assembly checkpoint genes BUB3, MAD2L1, and genes involved in DNA replications MCM2, MCM6 to be downregulated in response to fludarabine phosphate treatment. Similarly, we found the expression of PGK1, whose metabolic activity is regulated by MYC [62], to be downregulated upon fludarabine phosphate administration. These results suggest that fludarabine phosphate affects N-MYC-induced transcriptional reprogramming in NEPC cells.

### 3.4. Enhanced ROS Production Destabilizes N-MYC Protein by Inhibiting AKT Signaling and Is Responsible for the Reduced Survival of NEPC Cells

**Figure 4:**
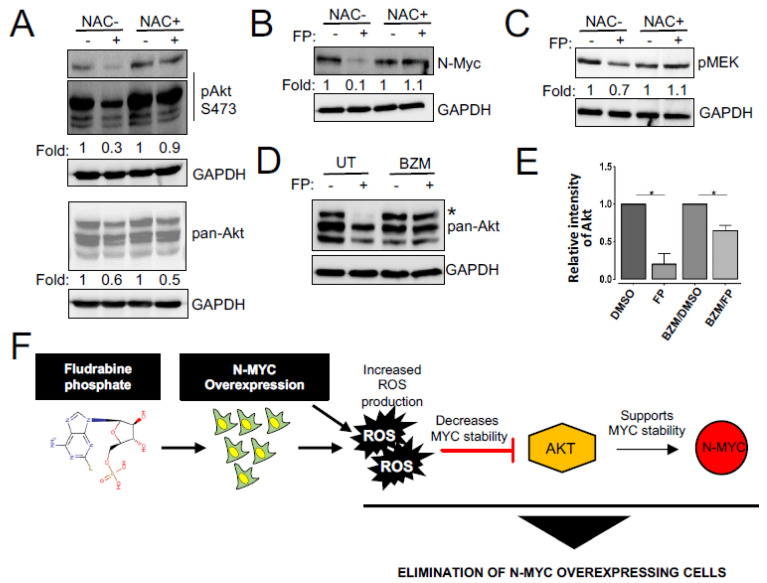
Fludarabine phosphate destabilizes N-MYC protein by inhibiting AKT signaling. (**A**) Fludarabine phosphate inhibits AKT activation. LASCPC-01 cells were pretreated with the ROS scavenger, NAC for 3 h, then treated with fludarabine phosphate at 25 μM for 48 h. Whole-cell extracts were subjected to Western blot analysis for phospho Akt and Akt. Cell signaling antibodies AKT #9272, phospho-AKT Ser473 #9271 against AKT and phosphoAkt used in this study detect AKT1,2 and 3. For phospho-Akt, short exposure and long exposure are presented. (**B**) Fludarabine phosphate triggers NMYC downregulation. LASCPC-01 cells were treated as in (**A**) and whole-cell extracts were subjected to Western blot analysis for N-Myc and representation from one of the three biological replicates is presented. (**C**) Fludarabine phosphate effect on pMEK. LASCPC-01 cells were treated as in (**A**) and whole-cell extracts were subjected to Western blot analysis for pMEK and representation from one of the three biological replicates is presented. (**D**) Fludarabine phosphate targets Akt proteins for proteasome-mediated degradation. LASCPC-01 cells were pre-treated for three hours with bortezomib (BZM) at 5 nM or matching volume of solvent, then treated with fludarabine phosphate at 25 μM for 48 h. Whole-cell extracts were analyzed for total AKT levels by Western blotting. (**E**) Quantitation of Akt level (the upper band *) of the WB in figure E, from two biological replicates. ImageLab software was used to quantify the bands. Akt *p* values 0.0153 and 0.0198 for FP-treated and BZM/FP, respectively. (**F**) Schematic illustration of the effect of fludarabine phosphate on N-MYC overexpressing cells.

We next investigated the mechanism responsible for the effect of fludarabine phosphate on N-MYC protein levels. Previous studies have demonstrated that N-MYC degradation is negatively regulated by PI3K/AKT and Raf/MEK pathways [30,31] and that fludarabine phosphate may also affect AKT levels in an ROS-dependent fashion [63]. Therefore, we monitored phospho-AKT and phospho-MEK levels in fludarabine phosphate-treated NEPC cells. In line with previously published observations, we found total and phospho-AKT levels decreased upon fludarabine phosphate application, while treatment with the free-radical scavenger, *N*-acetyl-L-cysteine (NAC) preserved phospho-AKT, which is responsible for active signaling (Figure 4A). This indicates that the effect of fludarabine phosphate on AKT signaling is dependent on ROS production and in agreement, while we observed a decrease in N-MYC protein level when the LASCPC-01 cells were treated with fludarabine phosphate, N-MYC was stabilized in the presence of NAC (Figure 4B). Interestingly, fludarabine phosphate treatment did not cause any major impact on phospho-MEK levels (Figure 4C). While dual blocking of PI3K and MEK pathways is toxic to most cells, including normal tissue, we found that fludarabine phosphate treatment mainly affected the AKT pathway in an ROS-dependent fashion. Thus, the effect of fludarabine phosphate on N-MYC protein downregulation is most likely dependent on the inhibition of the PI3K/AKT pathway. To examine if fludarabine phosphate triggered AKT degradation via the proteasome, we monitored AKT protein levels in cells treated with fludarabine phosphate in the presence and absence of the proteasome inhibitor, bortezomib (BZM). These experiments showed that bortezomib treatment prevented AKT degradation and preserved its levels (Figure 4D,E). In summary, the effects of fludarabine phosphate on AKT and N-MYC appeared to be a consequence of increased ROS, and this is likely to provide a treatment advantage (Figure 4F).

**Figure 5:**
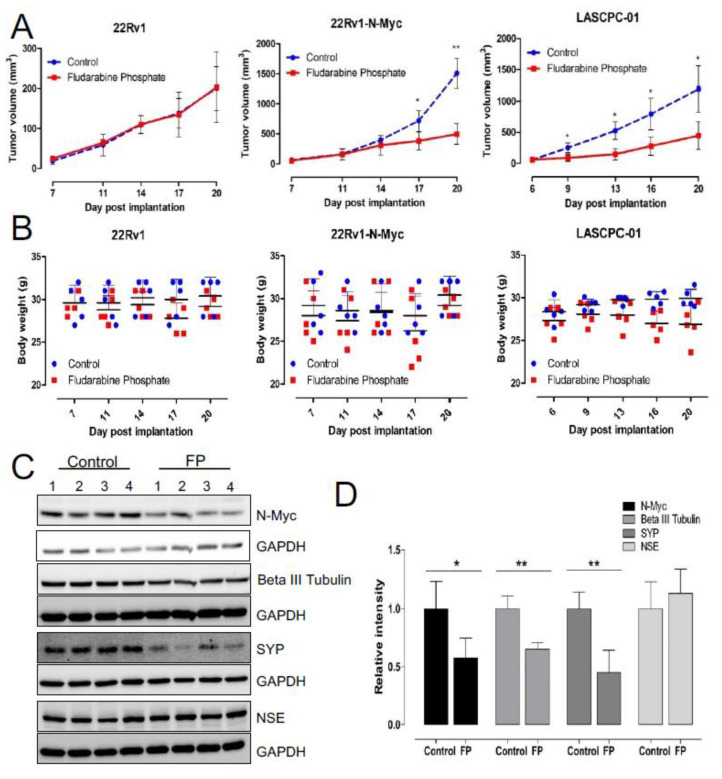
Fludarabine phosphate reduces tumor growth in NEPC xenograft models. (**A**) Validation of fludarabine phosphate treatment in 22Rv1, 22Rv1-N-Myc and LASCPC-01 xenograft models. Each cell line was implanted at 1 × 10^6^ cells subcutaneously by injection in PBS: Matrigel mixture at the 1: 1 ratio of in a total volume of 100 μL and monitored for tumor development. Fludarabine phosphate or DMSO was administered through i.p. injections at 120mg/kg starting day 6–7 post cell lines engraftment. Total of 10 i.p. injections were given per mouse (one i.p. injection per mouse per day was given for five consecutive days followed by two days rest and then, treatment continued for another five i.p. injections over five days). Tumors were measured every 3–4 days using digital caliper, and tumor volume was calculated using the formula A/2*B2 where A and B represent the long and short diameter of the tumor, respectively. Two biological experiments were performed n = 2 with 4 to 5 mice per group and the graph represents one biological replicate. (* *p* < 0.05; ** *p* < 0.01). (**B**) Graphs show body weights of fludarabine phosphate treated and DMSO-control mice. Mice were weighed every 3–4 days to assess potential side effects of the fludarabine phosphate administration. (**C**) Fludarabine phosphate reduces N-Myc protein levels in tumor tissue. Upon experiment termination, tumor tissues were collected from the LASCPC-01 xenograft model, tumor tissue homogenates were prepared, and Western blot analysis were performed for the indicated proteins in individual tumors. Western blot analysis was performed from individual tumor samples. (**D**) Quantitation of Western blots (* *p* < 0.05; ** *p* < 0.01) (**C**), Image Lab software was used to quantify the bands.

While our investigation has identified fludarabine phosphate as a potential FDA-approved drug that can preferentially inhibit N-MYC overexpressing cells in vitro, we next examined if its application in xenograft models will reduce tumor growth. To test this, subcutaneous xenograft tumors were generated in experimental animals by injecting LASCPC-01 cells in immunodeficient male NOD.Cg-*Prkdc^scid^ Il2rg^tm1Wjl^*/SzJ (NOD-SCID) mice (Supp. Figure S3A). In addition, to eliminate cell line specific effects, we also used an independent, previously described isogenic model 22Rv1-Parental (22-P) and 22Rv1-N-Myc-overexpressing (22-Myc), where the differential expression of N-MYC has been previously shown to develop poorly differentiated, invasive prostate cancer that is molecularly similar to human NEPC [13]. Treatment of these animals with fludarabine phosphate was initiated when tumors reached a detectable size. Fludarabine phosphate or DMSO was administered through intraperitoneal (i.p.) injections at the dose of 120 mg/kg per injection, as previously described [64]. This dosage was selected based on the dosage used in multiple previous publications [64,65,66,67]. A total of 10 i.p. injections were given per mouse (one injection per day for five days) followed by two days of rest and then, continued as one injection per day again for another five days, as previously reported [64], and the animals were monitored for 20 days (Supp. Figure S3A). This experiment revealed that matching our tissue culture observations, treatment with fludarabine phosphate, selectively suppressed the growth of N-MYC overexpressing prostate cancer tumors (Figure 5A). The treated and control mice were also monitored for body weight and no adverse effects, such as a decrease of more than 10% in body weight, were encountered (Figure 5B). Tumor samples were subjected to Western blot analyses and tumor sections were stained with antibodies for neuroendocrine markers. Interestingly, consistent with in vitro studies, decreased MYC protein levels were observed in tumors treated with fludarabine phosphate (Figure 5C,D). In addition, these analyses also showed a decrease in SYP and β-tubulin III levels in Western blots alone (Figure 5C,D), but no major reduction in neuronal markers was observed by immunohistochemistry (Supp. Figure S3B) probably because of the lower sensitivity of this assay. Taken together, our findings demonstrate that fludarabine phosphate reduces N-MYC protein levels in NEPC cells in vivo and selectively suppresses *MYCN*-overexpressing tumors.

## 4. Discussion

There is a pressing need for continued research in rare conditions, such as NEPC, as these disorders are often overlooked due to the costs involved with discovering therapeutic agents. High-throughput screens for therapeutic repurposing can be a viable option for these rare diseases, as initial associated costs are dramatically reduced in these approaches, where FDA-approved drugs have already gone through clinical trials. High-throughput screens can also be effective in drug-resistant contexts, whereby the mechanism of resistance is unknown or not suitable for direct pharmacological targeting. Since most FDA-approved drugs have been extensively characterized, hits that are identified in high-throughput screens may provide both mechanistic insights and represent good therapeutic agents ready for clinical use.

NEPC is a condition that arises from androgen deprivation of prostate adenocarcinoma. While this condition affects only a smaller population, it results in the death of approximately 98% of patients within a year of diagnosis. With the expected rise in the incidence of prostate cancer and the use of stronger antiandrogens, the rise of NEPC is expected to rise, and unfortunately, there is no treatment available to date. NEPC has been shown to overexpress *MYCN*, which is a known proto-oncogene that is not typically expressed in adult tissue. *MYCN* is also associated with tumors arising from the neural system and is commonly expressed in all forms of neuroendocrine cancers. Here, we identified multiple FDA-approved molecules including fludarabine phosphate that are selectively effective against *MYCN* overexpressing NEPC cells. In the case of fludarabine phosphate, this selectivity may be due to its ability to inhibit AKT signaling in an ROS-dependent manner, ultimately reducing the abundance of the N-MYC protein in N-MYC-addicted NEPC cells. Interestingly, we did not observe changes in N-MYC levels in the 22-P/22-MYC isogenic model (data not shown), although our experiments showed that N-MYC overexpressing cells inherently produce higher ROS (Supp. Figure S3C) and following fludarabine phosphate administration, ROS level increased even further in these cells (>2-fold; *p* < 0.05) (Supp. Figure S3D). This is not entirely surprising, as elevated ROS levels can affect multiple components of the molecular machinery in addition to AKT and N-MYC [68,69], and indicates that while the selective character of fludarabine phosphate action remains consistent, molecular mechanisms underlying this selectivity may be context-dependent. In summary, our study suggests that increasing ROS by treatment with fludarabine phosphate is advantageous for eliminating N-MYC-overexpressing NEPC cells most likely because higher N-MYC levels also enhance ROS production. The resulting high ROS accumulation accentuates the lethal effect of fludarabine phosphate by causing DNA damage, while also reducing N-MYC abundance by blocking its AKT-mediated phosphorylation. All this culminates in the apoptotic cell death in N-MYC-addicted cancer cells. Fludarabine phosphate also reduces AKT levels in an ROS-dependent fashion, which is likely to support cell elimination. Overall, this provides a mechanistic rationale for why, compared to cells without *MYCN* expression, N-MYC overexpressing cells are preferentially sensitive to fludarabine phosphate treatment (Figure 4F).

Previous studies have in fact shown that a brief suppression of N-MYC is associated with an irreversible change in the cellular program [70] and a two-fold decrease in oncogenic levels of MYC was sufficient to induce tumor regression [71]. As our results indicate that fludarabine phosphate reduces N-MYC levels, we believe that this should also support tumor regression. That said, fludarabine phosphate treatment in chronic lymphocytic leukemia (CLL) patients has been shown to affect the MYC-specific regulatory network [72]. This study has shown that MYC and its targets are downregulated in patients that are sensitive to fludarabine phosphate. Interestingly, the same study has also reported that the Myc target gene network was upregulated in patient cells that were resistant to the fludarabine [72]. This indicates that CLL cells may re-wire and can overcome the effects of fludarabine by simply restoring the MYC-specific transcriptional reprogramming and that the irreversible change in the cellular program, by brief suppression of MYC [70] may not be applicable to CLL patients. Thus, while our work indicates that fludarabine phosphate might trigger N-MYC downregulation, to sustain this effect and overcome residual resistance to fludarabine, the development of complementary combination therapies would be imperative. From this perspective, our work has identified several additional potential compounds such as daunorubicin or chidamide, and histone modulators such as ciclopirox or belinostat [49,50,51,52] that are also known to affect MYC (Supp. Table S1). Combination strategies with these drugs may provide sustainable N-MYC suppression and may benefit the treatment of *MYCN* overexpressing tumors.

Currently, the primary use for fludarabine phosphate is in the treatment of leukemia, specifically CLL and prolymphocytic leukemia, as well as non-Hodgkin’s lymphoma. However, it has also been shown to be effective in treating acute myeloid leukemia, hairy cell leukemia, cutaneous T-cell lymphoma, and Waldenstrom’s macroglobulinemia, as well as a regimen for pre-allogenic bone marrow transplant [73,74]. It is important to note that in many of these cases, fludarabine phosphate in combination with cyclophosphamide is used for immune modulation therapies using CART cells. It is interesting to note that MYC expression can suppress the type I interferon (IFN) signaling pathway and de-repression of IFN regulator genes may potentiate the immunotherapy [75]. Accordingly, we suspect the application of fludarabine phosphate can downregulate MYC protein levels and may possibly derepress IFN signaling in NEPC, a notion that is yet to be tested. Thus, while fludarabine phosphate might negatively regulate AKT signaling, its additional role in immune modulation, possibly via MYC downregulation, may provide an added advantage for combination therapies.

## Data Availability

The RNA seq data generated in this study are deposited in Gene Expression Omnibus (GEO) database and can be obtained with the accession number GSE181119.

## Supplementary Materials

The following supporting information can be downloaded at: https://www.mdpi.com/article/10.3390/cells11142246/s1, Figure S1: Details of drug library screened; Figure S2: Enrichment analyses; Figure S3: Details of Xenograft studies; Figure S4: Raw images of western blots; Figure S5: Raw images of western blots; Table S1: Information of the hits identified; Table S2: List of primers.
