# Supplementary material for: A Drug Repurposing Screen Identifies Fludarabine Phosphate as a Potential Therapeutic Agent for N-MYC Overexpressing Neuroendocrine Prostate Cancers
Source: Cells. 2022 Jul 20;11(14):2246. doi: 10.3390/cells11142246 (PMC9317991; doi:10.3390/cells11142246)
Supplement: Supplementary file 1 [file cells-11-02246-s001.zip › cells-1748945-supplementary.pdf]

A

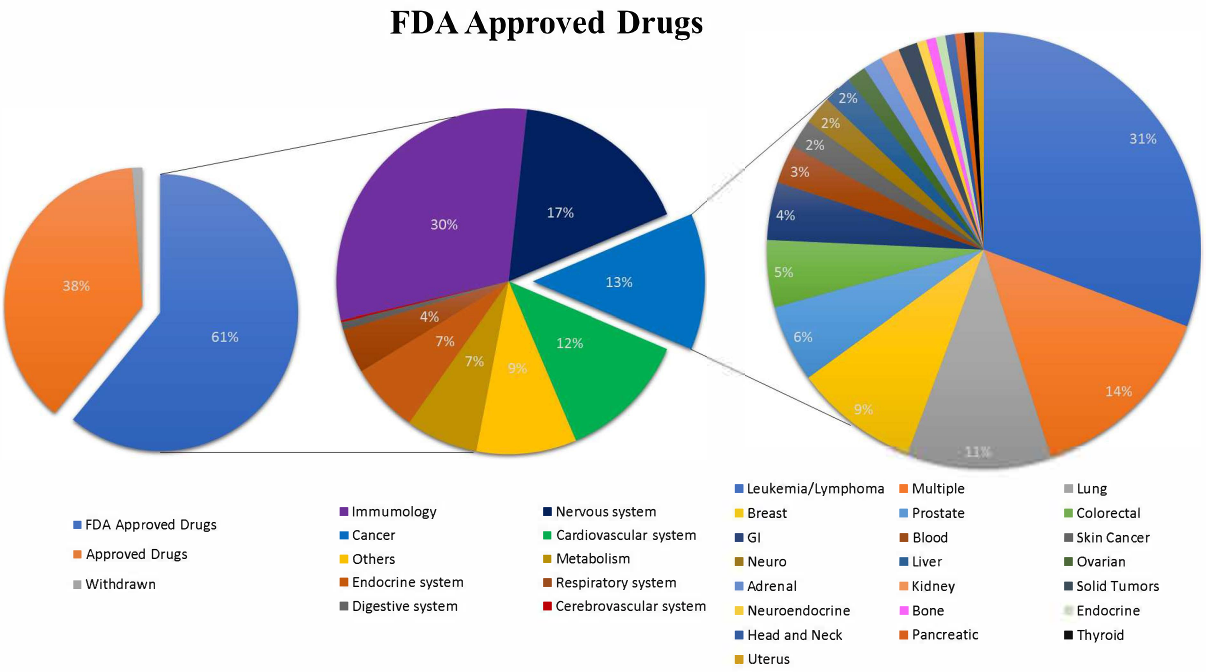

B

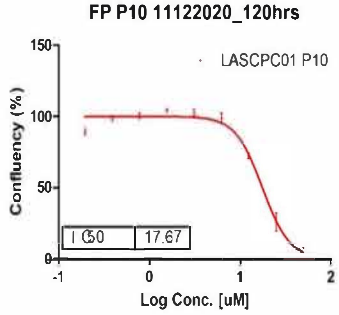

Supp. Figure S1: (A) The Pie chart shows the Targetmol Drug Library composed of 1813 small molecule compounds. FDA approved drugs compose 61% (1,105 molecules) clinically available in the United States, while 38% approved drugs (687 molecules) are clinically used outside of the United States. The withdrawn small molecules (21 molecules) were previously used in United States but have been replaced due to new advancements in treatment options or severe adverse effects.(B) IC50 values for Fludarabine phosphate in the NEPC cell lines. LASCPC-01 cells were treated with Fludarabine phosphate at the indicated concentrations (x-axis) and cells confluence (y-axis) was monitored over the course of five days. All values are averages of replicates expressed relative to the values of DMSO-treated cells normalized to 100%.

A

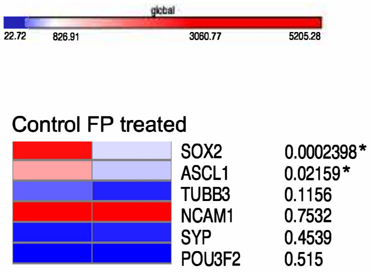

B

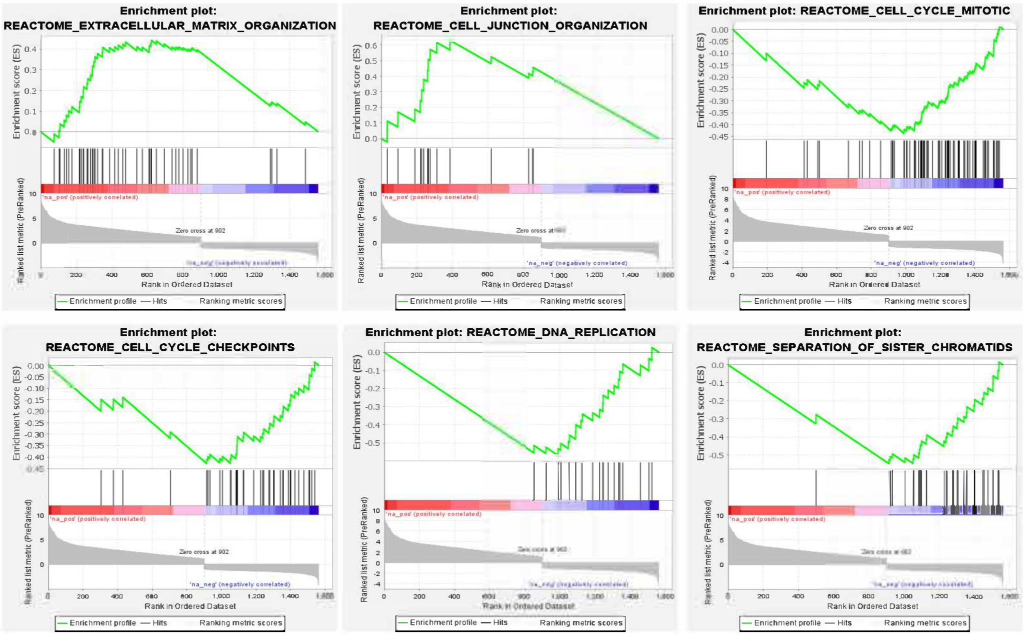

Supp. Figure S2: (A) RNAseq analyses in LASCPC-01 cells (with and without Fludarabine phosphate treatment) show the expression analysis of *SOX2, ASCL1* and the rest of NED markers at 48 hours of Fludarabine phosphate treatment compared to the DMSO control at 48 hours. p-value significance is presented beside each gene name. *represents those genes that are significant. The legend bar shows the range of expression with blue representing down regulated genes and red representing upregulated genes. (B) GSEA enrichment analyses for Reactome pathways for genes up/down regulated in Fludarabine phosphate treatment.

Supp. Figure S3

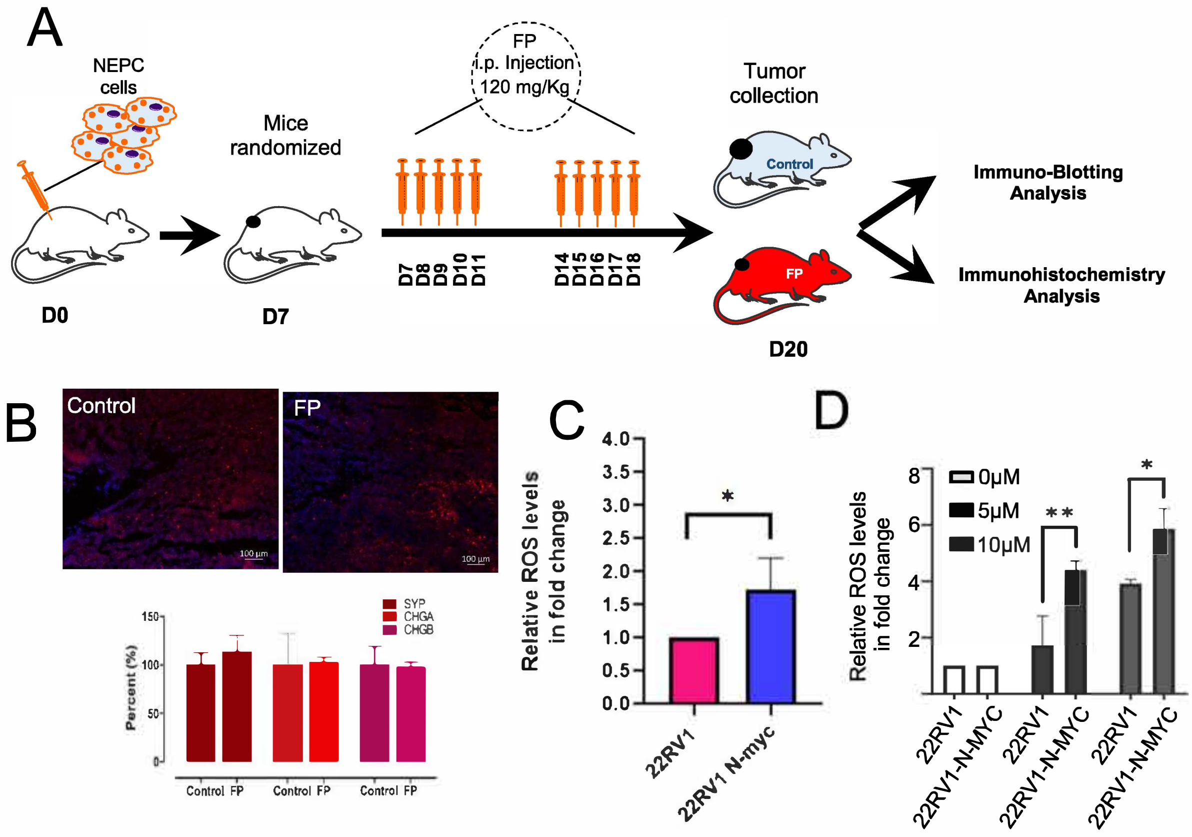

Supp. Figure S3: (A) Schematic illustration of the Fludarabine phosphate treatment in NEPC xenograft model. (B) A sample image of immunohistochemistry for SYP is shown. Quantitation of SYP, CHGA, and CHGB in tumor tissues are presented on the right. Tumor tissues were collected from the LASCPC-01 xenograft model. Tumor tissues from two mice were stained with the SYP, CHG A and CHG B; 50-81 images were analyzed of the two tumor tissues control and Fludarabine phosphate treated mice and calculated as a percent to the DAPI stained cells. All images were captured using an Axio Observer 7 inverted fluorescence microscope (Carl Zeiss). (C) Basal ROS levels estimated by DCFDA assay showing higher ROS levels in N-myc overexpressed cells as compared to parental cells. (D) Intracellular ROS levels were estimated by DCFDA assay showing higher ROS production in 22Rv1-N-Myc cells as compared to 22Rv1 cells when treated with FP using 5 µM and 10 µM concentrations. * ($p<0.05$) and ** ($p<0.002$).

Supp. Figure S4

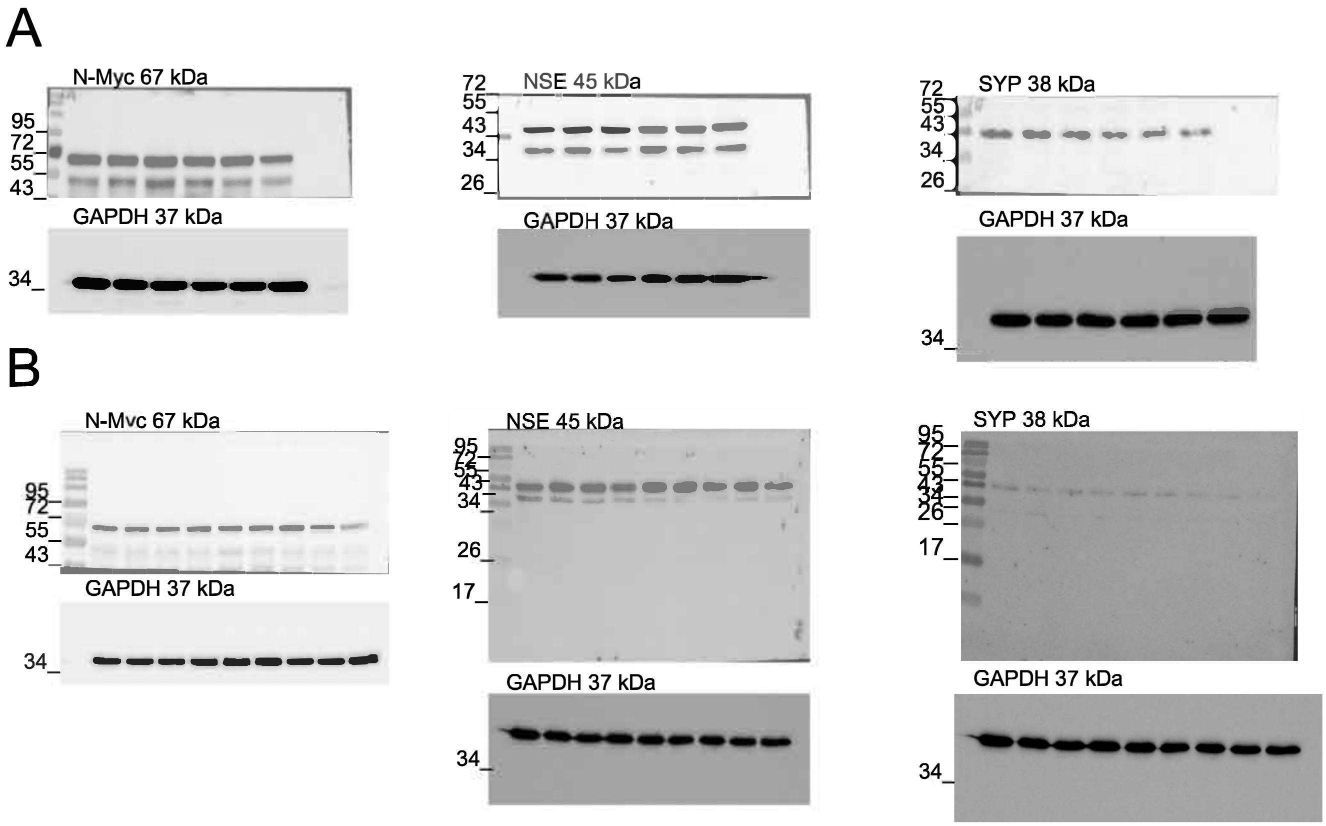

Supp. Figure S4: Full-length western blots pertaining to Fig. 3D (A). Full-length western blots pertaining to Fig. 3E (B).

Supp. Figure S5

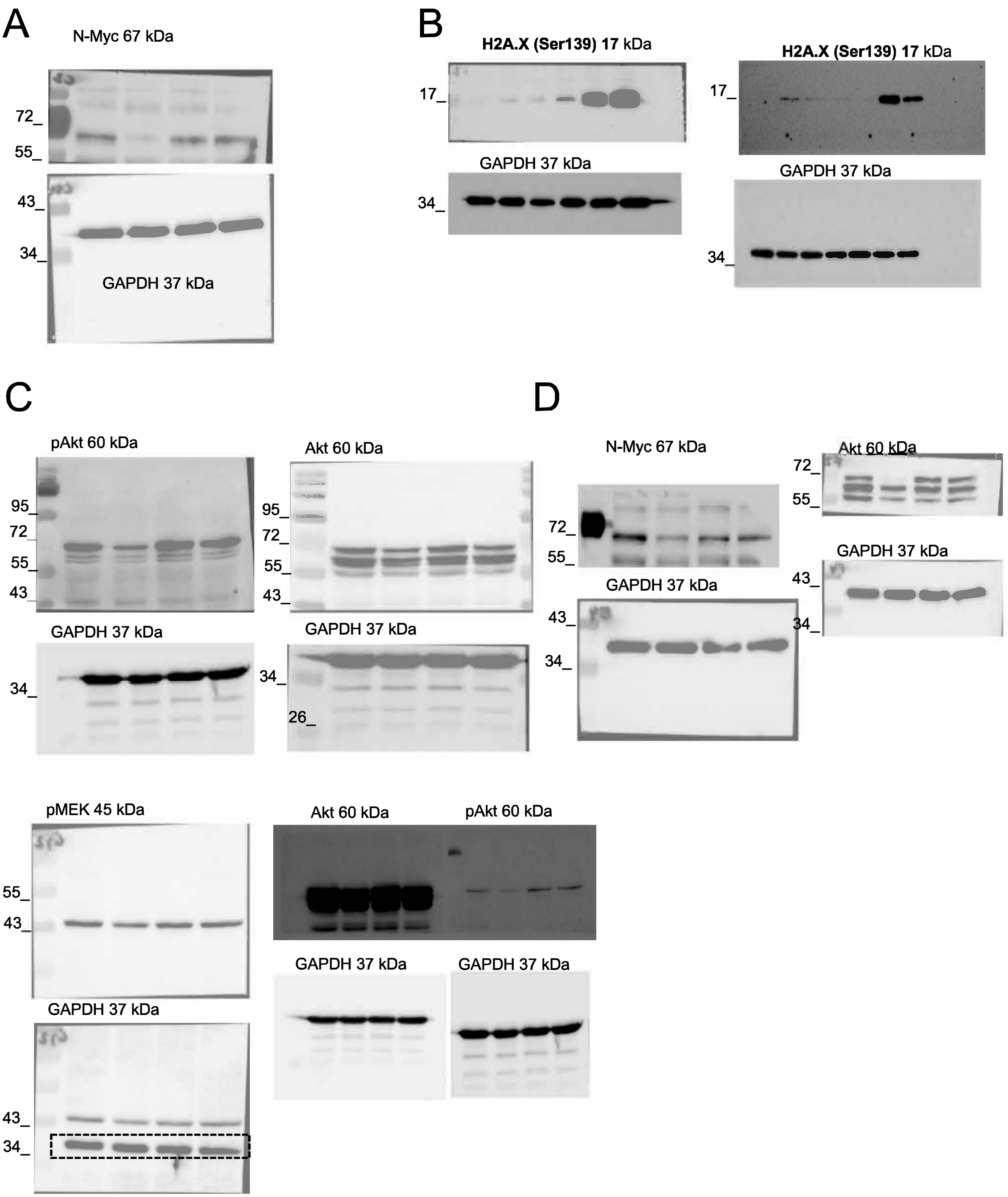

Supp. Figure S5: Full-length western blots pertaining to Fig. 4B (A), 3C (B,), and (4A and 4C) (C). Full-length western blots pertaining to Fig. 4D (D)

Supp. Table S1

DRUG	PURPOSE	TARGETS	MYC ASSOCIATION	PMID
**Fludarabine**	*chronic lymphocytic leukemia (CLL)<br>*Salvage therapy for non-Hodgkin's lymphoma<br>*Acute leukemia<br>*Hepatocellular Carcinoma	*STAT1, XIAP, IL7, IL7R, CCL3, MAX, CD6, RB1CC1, BRCA2, TGFB1,RAD23B<br>*Akt phosphorylation	* Inhibitory action at the transcriptional regulation of MYC.	26690614, 3078261, 32133897, 31011625, 24057147, 20487546.
**Fludarabine phosphate**	* Leukemia<br>* Non-Hodgkin Lymphoma (NHL)<br>*Lymphocytic B-Cell leukemia<br>*Prostrate cancer	* Purine nucleoside phosphorylase<br>* CD-4 Molecule<br>* Deoxyadenosine triphosphate	N/A	15005347, 15154738, 19283354, 15493036, 22009763, 15823705, 1699280.
**Pemetrexed disodium**	* Pleural mesothelioma<br>*Non-small lung carcinoma(NSCLC)	* Thymidylate synthase<br>* Dihydrofolate Reductase<br>* Tetrahydro-folate synthase	* In combination with gemcitabine inhibit the expression of the MYC	15497247, 12431831, 12907242, 24684846.
**Methotrexate**	* Non-Small-Cell lung cancer (NSCLC)<br>* Rheumatoid arthritis	*Nucleotide synthesis<br>* Dihydrofolate Reductase<br>* Thymidylate synthase<br>*Tetrahydro folate synthase	* Decreases the translation efficiency of c-myc	18353995, 19373092, 24284432, 11742712, 12907242.
**Daunorubicin hydrochloride**	* Glioblastoma<br>*Acute myeloid leukemia (combination with Cytarabine)	*Topoisomerase IIA	*Downregulates expression of MYC	2974416, 32955826, 20534341, 9523731.
**Chidamide**	* Peripheral T cell lymphoma (PTCL)<br>* Triple negative breast cancer (TNBC)	*Inhibits Histone Deacetylase(HDAC)<br>*JAK/STAT3 inhibitor	*Downregulates expression of C-myc	28298231, 31257519, 20060381, 31907371, 27508038.
**Cliclopirox**	*Colon cancer<br>*Onchomycosis<br>*Mycoses	Eukaryotic translation initiation factor 5a.	*Myc signalling pathway	25821426, 33772895, 33573561, 28684529.
**Belinostat (PDX101)**	* Peripheral T-cell lymphomas (PTCL)	* Inhibits Histone deacetylase(HDAC)	*In combination with Volasertib results in marked reduction of c-myc<br>*In RMS RD and RH 30 cell line downregulate the expression of c-myc protein.	26101246, 28416758, 31325529.
**Crystal violet**	* Neoplasms<br>*Chagas disease	* Caspase-3<br>* Polyamine Oxidase 1<br>* Akt Serine/Threonine Kinase 1<br>*Inhibit proline transporter TcAAAP069		26613989, 31961864, 26177467, 18849857,
**Pemetrexed Disodium Hydrate**	*Lung adenocarcinoma	*Folate receptor alpha.		34079760,
**Yohimbine hydrochloride**	*Erectile dysfunction<br>*Anxiety disorders<br>*Panic disorder	*Adrenoreceptor Beta<br>*Amylase alpha 1b<br>*Prolactin		21856216, 22116378, 24972729, 23939512, 2570579, 3963600.

Supp. Table S1: The table shows several potential molecules that were identified in our screen using the Targetmol Drug library screen.

Supp. Table S1 Cont'd

DRUG	PURPOSE	TARGETS	MYC ASSOCIATION	PMID
**Bleomycin Sulfate**	* Pulmonary Fibrosis<br>* Warts	* Procollagen-proline 4-Dioxygenase<br>* Myeloperoxidase<br>* Elastin		31819797, 24231620, 1711753, 6179601, 27402190, 10556151.
**Carfilzomi (PR-171)**	* Multiple Myeloma<br>*Myeloproliferative Disorders<br><br>* Neoplasms	* Proteasome Endopeptidase Complex<br>* Histone Deacetylase<br>* Chymotrypsin<br>* AMPKα Phosphorylation		31341235, 33809268, 33848640, 33735504, 28243125, 22929803, 30482794.
**Tanshinone I**	* Cardiovascular Diseases<br>* Neoplasms<br>* Cerebrovascular Disorders<br>*Ovarian cancer	* Ferruginol Synthase<br>* Bcl2 Apoptosis Regulator<br>* Caspase-3<br>*PI3K/Akt/mTOR pathway		33236791, 33617968, 31820522, 31704239, 31820522.
**Buflomedil hydrochloride**	*Peripheral Vascular Diseases<br>* Cerebrovascular Disorders<br>* Ischemia	*Phosphopyruvate Hydratase<br>*U-Plasminogen Activator<br>* Carbonic Anhydrase 1		9482510, 16149710, 7705991, 20452806.
**Arsenic oxide (3)**	* Ovarian cancer<br>*Acute promyelocytic leukemia	* Peripheral myelin protein 2<br>* Pds5 Cohesin Associated factor B<br>* Inorganic Phosphate Transporter 1-2<br>*PI3K,Akt, mTOR Pathway.		31257975, 30803522, 29856616, 34047865, 25157412, 30225639.
**Iodoquinol**	* Acrodermatitis Enteropathica<br>* Amebiasis<br>* Dysentery	* Sulfotransferase Family 1c Member 4<br>*Sulfotransferase Family 1a Member 2<br>*Sulfotransferase Family 1b Member 1		53650, 8433407, 226725, 27449410.
**Salifungin**	* Dermatitis<br>* Candidiasis<br>*SARS-CoV-2	* SARS-CoV-2 S protein		6019797, 6581915, 33324406.
**Amorolfine hydrochloride**	* Biliary tract carcinomas (BTCs)<br>*Onchomycosis<br>*Pythium insidiosum infections	* Ergosterol synthesis pathway<br>*Arachidonate 5-Lipoxygenase<br>*Beta-Glucuronidase<br>*Lysozyme		31018139, 30925734, 32400872, 16964330, 1840158, 2599717.
**Iodoquinol**	* Acrodermatitis Enteropathica<br>* Amebiasis<br>* Dysentery	* Sulfotransferase Family 1c Member 4<br>*Sulfotransferase Family 1a Member 2<br>*Sulfotransferase Family 1b Member 1		53650, 8433407, 226725, 27449410.

Supp. Table S1 (Cont'd): The table shows several potential molecules that were identified in our screen using the Targetmol Drug library screen.

DRUG	PURPOSE	TARGETS	MYC ASSOCIATION	PMID
**Salifungin**	* Dermatitis<br>* Candidiasis<br>*SARS-CoV-2	* SARS-CoV-2 S protein		6019797,<br>6581915,<br>33324406.
**Amorolfine hydrochloride**	* Biliary tract carcinomas (BTCs)<br>*Onchomycosis<br>*Pythium insidiosum infections	* Ergosterol synthesis pathway<br>*Arachidonate 5-Lipoxygenase<br>*Beta-Glucuronidase<br>*Lysozyme		31018139,<br>30925734,<br>32400872,<br>16964330,<br>1840158,<br>2599717.
**Irinotecan hydrochloride trihydrate**	* Colorectal cancer<br>*Advanced solid tumors (Breast cancer, pancreatic cancer and Gastric cancer)<br>*High grade gliomas	* Topoisomerase I		314952238,<br>27871319,<br>33924355,<br>16343744.
**Triethylenethiophos-phoramide**	* Brain tumor and adult malignant lymphoma<br>*Colorectal carcinoma.	* Cytochrome P450 2B6<br>*Cd34 Molecule<br>*Colony Stimulating Factor 3		31428821,<br>4960423,<br>11950782,<br>2316007,<br>7536025.
**Irinotecan**	* Small cell lung cancer<br>*Advanced gastric and esophageal adenocarcinoma<br>*Cervical cancer<br>*Neuroblastoma	* Topoisomerase I inhibitor<br>*PI3K/Akt Signalling		33074323,<br>16416165,<br>12109804,<br>23377825,<br>32664667,<br>30415007.
**Arteether**	* Malaria<br>*Breast cancer<br>*Oral squamous carcinoma	* Unspecific Monooxygenase,<br>*Cytochrome P450<br>* Alkaline Phosphatase		286892553,<br>24076591,<br>17163469,<br>23391499,<br>18350255,<br>16637400.
**Hydroxyquinoline**	* Wilson's disease<br>*Alzheimer's disease (in combination with Donepezil + Proparglyamine)			29407674,<br>24813882.
**1,2Dipheylhydrazine**	*Carcinogenesis	* Pyranose Oxidase<br>*Neurexophilin2<br>*Aldos-2-Ulose Dehydratase.		10798712,<br>8352649,<br>18361375,<br>8352649
**Ciclopirox ethanolamine**	* Chronic hepatitis B virus (HBV) infection<br>*Fungal and yeast infections *Promyeloid leukemia	*RNA and DNA		31097716,<br>15271193,<br>190022.
**Oxethazaine**	* Chronic hepatitis B virus (HBV) infection.	*Calcium-signalling Pathway.		26838678
**Lauroseptol**	* Neoplasms<br>*Triple negative breast Cancer (TNBC).	* PI3K/Akt pathway.		32878794,<br>28810532,<br>34013357.
**Lacidipine**	* Hypertension<br>* ovarian cancer	* Calcium channels<br>* NANOG, SOX2, and CD133.		14524737,<br>32230901.
**Tolcapone**	* Parkinson disease<br>* Neuroblastoma	* Catechol-O-methyl transferase Inhibitor.<br>*Reduces ATP production in the neuroblastoma cells.		32809428,<br>28429453.

Supp. Table S1 (Cont'd): The table shows several potential molecules that were identified in our screen using the Targetmol Drug library screen.

Supp. Table S2

List of primers sequences used in RT-qPCR analysis

Gene symbol	Primer (5' – 3')
**MYCN**	Forward - ACCACAAGGCCCTCAGTACCTC
	Reverse - TGACAGCCTTGGTGTTGGAGGA
**GAPDH**	Forward - GTCTCCTCTGACTTCAACAGCG
	Reverse - ACCACCCTGTTGCTGTAGCCAA
**RPLP**	Forward - AGCCCAGAACACTGGTCT
	Reverse - ACTCAGGATTTCAATGGTGCC
